# The super-resolution reconstruction algorithm of multi-scale dilated convolution residual network

**DOI:** 10.3389/fnbot.2024.1436052

**Published:** 2024-08-16

**Authors:** Shanqin Wang, Miao Zhang, Mengjun Miao

**Affiliations:** ^1^School of Information Engineering, Chuzhou Polytechnic, Chuzhou, China; ^2^School of Computer, Qinghai Normal University, Xining, China

**Keywords:** super-resolution reconstruction, convolutional neural network, dilated convolution, multi-level features, residual dense block, attention channel

## Abstract

Aiming at the problems of traditional image super-resolution reconstruction algorithms in the image reconstruction process, such as small receptive field, insufficient multi-scale feature extraction, and easy loss of image feature information, a super-resolution reconstruction algorithm of multi-scale dilated convolution network based on dilated convolution is proposed in this paper. First, the algorithm extracts features from the same input image through the dilated convolution kernels of different receptive fields to obtain feature maps with different scales; then, through the residual attention dense block, further obtain the features of the original low resolution images, local residual connections are added to fuse multi-scale feature information between multiple channels, and residual nested networks and jump connections are used at the same time to speed up deep network convergence and avoid network degradation problems. Finally, deep network extraction features, and it is fused with input features to increase the nonlinear expression ability of the network to enhance the super-resolution reconstruction effect. Experimental results show that compared with Bicubic, SRCNN, ESPCN, VDSR, DRCN, LapSRN, MemNet, and DSRNet algorithms on the Set5, Set14, BSDS100, and Urban100 test sets, the proposed algorithm has improved peak signal-to-noise ratio and structural similarity, and reconstructed images. The visual effect is better.

## 1 Introduction

Single Image Super-Resolution (SISR) is a pivotal image processing technique within the field of computer vision. It finds widespread application in various domains such as satellite remote sensing (Yue et al., 2023; Zhao et al., 2023), medical imaging (Qiu et al., 2023; Wang et al., 2023), and facial recognition (Klemen and Vitomir, 2020; Hou et al., 2023), with its primary objective being to reconstruct a high-resolution (HR) image from its corresponding low-resolution (LR) counterpart.

Currently, single-image super resolution reconstruction techniques are categorized into three types: interpolation-based methods (Wang et al., 2023; Wu et al., 2023), reconstruction-based methods (Fu et al., 2023; Zhang et al., 2023), and learning-based methods (Zhang et al., 2020; Li et al., 2021, 2023; Zhou et al., 2021; Min et al., 2023; Zhao et al., 2023). Among these, due to the significant potential demonstrated by deep learning in the realm of computer vision, learning-based super-resolution algorithms have emerged as the dominant research direction. In 2014, Dong et al. (2014) first applied deep learning to super-resolution reconstruction, introducing a super-resolution algorithm using convolutional neural networks (Super-Resolution Convolutional Neural Network, SRCNN), achieving end-to-end learning. However, due to conducting only three convolutional operations, this algorithm was limited in the amount of image information it could extract. Addressing this issue, Chao et al. (2015) later proposed a fast convolutional neural network-based super-resolution reconstruction algorithm (Fast Super-Resolution Convolutional Neural Network, FSRCNN), which employed a deconvolution layer instead of bicubic interpolation in the upsampling process, and deepened the network from 3 layers to 8 layers. Following this, many researchers have dedicated efforts to developing algorithms with improved reconstruction outcomes. Timofte et al. (2017) introduced a super-resolution reconstruction algorithm based on a deep residual neural network (Accurate Image Super-Resolution Using Very Deep Convolutional Networks, VDSR), which incorporated the concept of residuals into SR, reducing the complexity of the network and allowing it not only to capture local features but also to grasp more global characteristics. However, as the network’s depth increased, there was a decrease in training speed. In response to this, Lim et al. (2017) presented an enhanced deep residual network-based super-resolution algorithm (Enhanced Deep Residual Networks for Single Image Super-Resolution, EDSR), which, by eliminating the BN (Batch Norm) layer, accelerated network convergence. Subsequently, with the advent of the generative adversarial network framework, Ledig et al. (2017) proposed a super-resolution reconstruction algorithm based on generative adversarial networks (Super-Resolution Generative Adversarial Network, SRGAN), incorporating this framework into SR to utilize perceptual loss and adversarial loss as the loss functions, thereby rendering the reconstruction results more lifelike. Li et al. (2018) proposed a multi-scale residual network-based super-resolution algorithm (Multi-scale Residual Network for Image Super-Resolution, MSRN), which leverages multi-scale feature fusion and local residual learning to fully exploit the features of images.

Despite the considerable reconstruction results achieved by the aforementioned deep learning-based image super-resolution algorithms, there remain several issues. Most of these algorithms attempt to improve reconstruction outcomes by increasing the network’s width and depth but struggle to extract deep-layer image information. Moreover, as the network deepens, problems such as the loss of high-frequency information and increased training time emerge during the computation process of each layer of the network. Additionally, for super-resolution algorithms, extracting complete and rich feature information from low-resolution (LR) images is crucial. Treating the extracted features from each channel equally limits the network’s expressive capability and fails to adequately highlight details such as image edges and textures.

To address the above issues, this paper proposes a multi-scale dilated convolution residual network, which mainly includes residual attention dense block and multi-scale residual module. Initially, an Residual Attention-Dense Block (RADB) is designed, composed of a densely connected block and a channel attention block, which can fully learn the features of the original low resolution image. Subsequently, on top of the RADB, a Dilated Multi-Scale Residual Module (DMRM) is constructed, capable of extracting more scales of low-resolution image information, improving the problem of small receptive fields, and enhancing cross-channel learning capability, thus better integrating extracted multi-scale features. Finally, a multi-level dilated convolution residual network based on dilated convolution is constructed through residual nesting, addressing the loss of significant detail information after multi-layer transmission and aiding in gradient flow. Moreover, sub-pixel convolution was employed for upsampling to reduce the complexity of the network.

Our contributions can be summarized as follows:

1. We propose a multi-scale dilation convolution residual network for image super-resolution, which learns the mapping relationship between low resolution images and high-resolution images and achieves good results in image super-resolution task.
2. To address the insufficient extraction of high-frequency information in images, we designed a Residual Attention-Dense Block (RADB) to learn features from the original low-resolution images. This enhances the network’s ability to discern and learn both high and low-frequency information from low-resolution images.
3. To address the limitations of convolutional receptive fields and the issue of potential information loss when extracting features through a single channel, we designed a Dilated Multi-Scale Residual Module (DMRM) based on dilated convolutions on top of the RADB. This module aims to extract multi-scale information from low-resolution images while preserving the integrity of high-frequency information.
4. Extensive experiments have shown that our method performs well in image super-resolution task.

## 2 Related word

### 2.1 Dilated convolution

Dilated convolution was initially utilized for semantic segmentation, where it demonstrated notable effectiveness in practical applications and was subsequently adopted across various domains within computer vision. Chen et al. (2014) are among the first to apply the concept of dilated convolution to address issues in image segmentation. Common image segmentation algorithms typically employ pooling and convolutional layers to increase the receptive field, which results in a reduction of the feature map dimensions. Subsequently, upsampling is used to restore the image size. This process of reducing then enlarging the feature maps decreases spatial resolution. Hence, there arises a need for an operation that can increase the receptive field while maintaining the size of the feature map, thereby substituting the roles of downsampling and upsampling operations.

**Figure 1:**
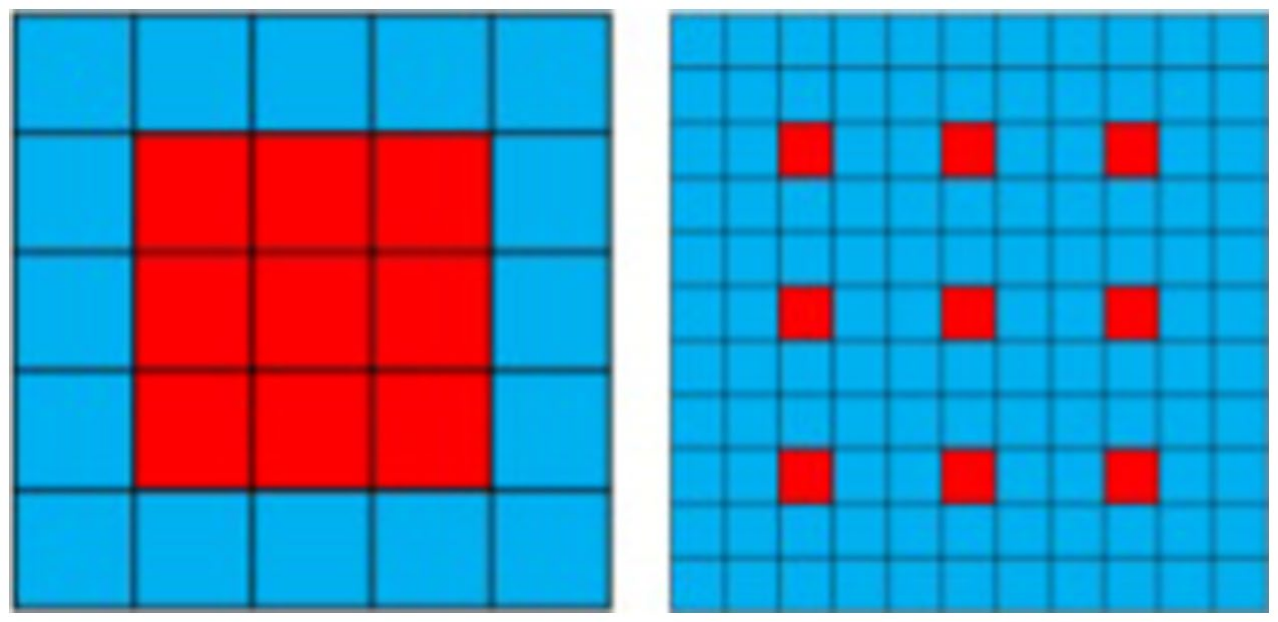
The difference between standard disclosure and empty disclosure (expansion rate = 3).

Unlike standard convolution, dilated convolution introduces a superparameter known as the “dilation rate,” which defines the spacing between each element of the convolutional kernel. By setting different dilation rates, the receptive field of dilated convolution varies, enabling the capture of multi-scale image information. This characteristic distinguishes the receptive field of standard convolution from that of dilated convolution (with a dilation rate of 3), The introduction of dilated convolution allows for broader contextual understanding without loss of detail, proving essential for enhancing detail and accuracy in tasks such as image segmentation and super-resolution as illustrated in Figure 1.

### 2.2 Channel attention

**Figure 2:**
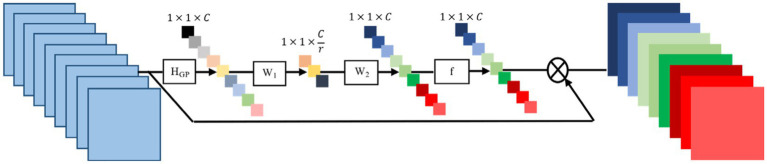
Channel attention mechanism.

In the process of image reconstruction, high-frequency information is vitally important. However, the majority of convolutional neural network-based methods for super-resolution image reconstruction treat the features in the channels equally, failing to distinguish between the low-frequency and high-frequency information across channels. Generating distinct attention for each channel’s features is a crucial step. Typically, convolutional layers have a limited receptive field and can only extract features within this field, unable to utilize the contextual information beyond it. Meanwhile, low-resolution images are rich in both low-frequency and high-frequency information; low-frequency information usually represents flatter areas, whereas high-frequency information is filled with edges, textures, and other details. For this purpose, global average pooling (Zhang et al., 2018) is utilized to transform the global spatial information within each channel into channel descriptors, by setting weights to denote the relevance between the channel and key information, as demonstrated in Figure 2. In this, $H_{GP}$ represents the process of adaptive average pooling, $W_{1}$ and $W_{2}$ represent the weights of the channel upsampling and downsampling layers respectively, and f denotes the operation of the Sigmoid function, 1×1×C represents the Height × Width × Channels, r represents the dimension compression ratio.

## 3 Proposed method

### 3.1 Overall network architecture

**Figure 3:**
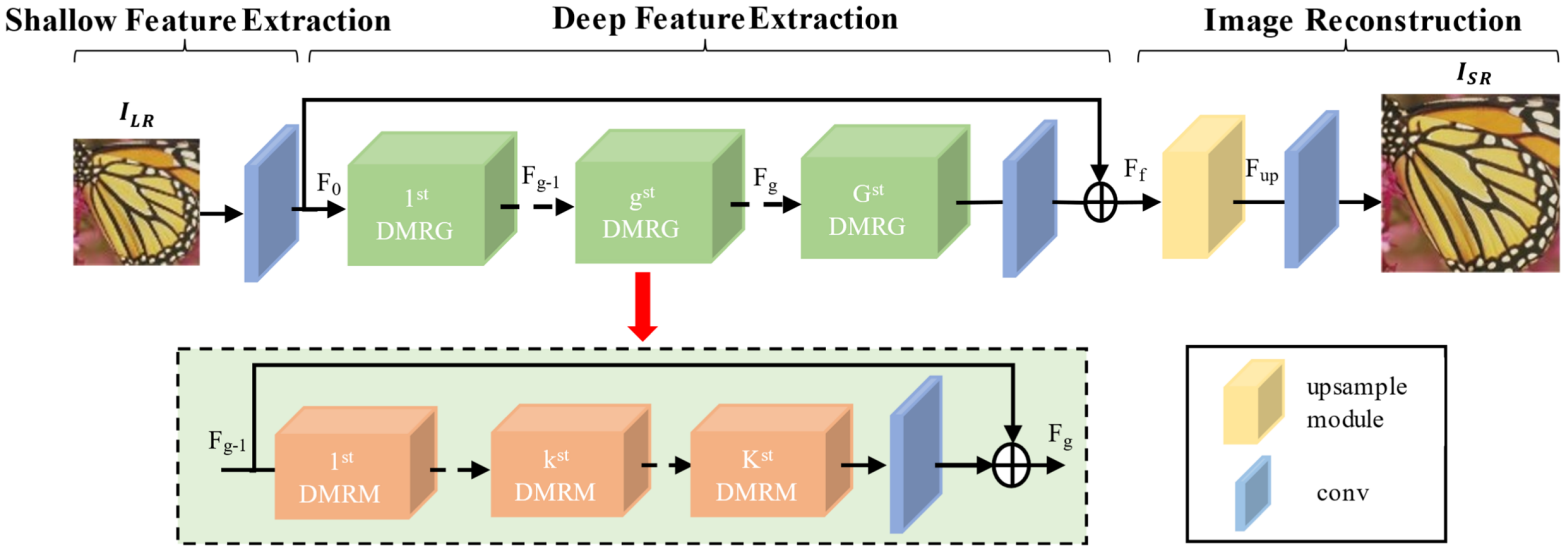
Multi-level attention network structure based on dilation convolution.

To address issues encountered in the image reconstruction process such as limited receptive field range, insufficient extraction of multi-level features, and the easy loss of image feature information, this paper designs a multi-level residual attention network based on dilated convolution. The schematic of this network framework is shown in Figure 3. The framework of the network consists of three parts: shallow feature extraction, deep feature extraction, and image reconstruction. The shallow feature extraction consists of a convolution layer with a 3×3 kernel; deep feature extraction is composed of 10 Dilated Multi-Scale Fusion Residual Groups (DMRG), each containing three dilated multi-Scale residual modules (See 2.2 for details) and one 3×3 convolution layer; and image reconstruction is made up of an upsampling module and a 1×1 convolution layer.

Assuming $I_{LR}$ and $I_{HR}$ represent the input low-resolution image and the reconstructed high-resolution image, respectively, initially, the initial convolution layer extracts the initial features $F_{0}$ from the low-resolution image, as shown in Equation 1:


(1) $$F_{0}=f_{0}\left(I_{LR}\right)$$

Here, $f_{0}$ denotes the operation of the initial convolution layer. Subsequently, deeper features are extracted from the initial features $F_{0}$ through multiple multi-scale fusion residual groups. The extracted deep features are then combined with the initial features through global residual connections to obtain the fused feature $F_{f}$, as shown in Equation 2:


(2) $$F_{f}=\left(F_{0}+f_{f}\left(D_{G}\left(\cdots \left(D_{g}\left(\cdots \left(D_{1}\left(F_{0}\right)\right)\cdots \right)\right)\cdots \right)\right)\right)$$

Here, $D_{G}$ represents the operation of the multi-channel fusion residual group, and $f_{f}$ represents the operation of the intermediate convolutional layer. Next, the upsampling module upsamples the fused features $F_{f}$, as shown in Equation 3:


(3) $$F_{up}=f_{up}\left(F_{f}\right)$$

Here, $f_{up}$ represents the upsampling operation, and $F_{up}$ represents the obtained upsampled features. Finally, the reconstruction convolutional layer $f_{rec}$ reconstructs the upsampled features, as shown in Equation 4:


(4) $$I_{HR}=f_{rec}\left(F_{up}\right)$$

### 3.2 Dilated multi-scale residual module

**Figure 4:**
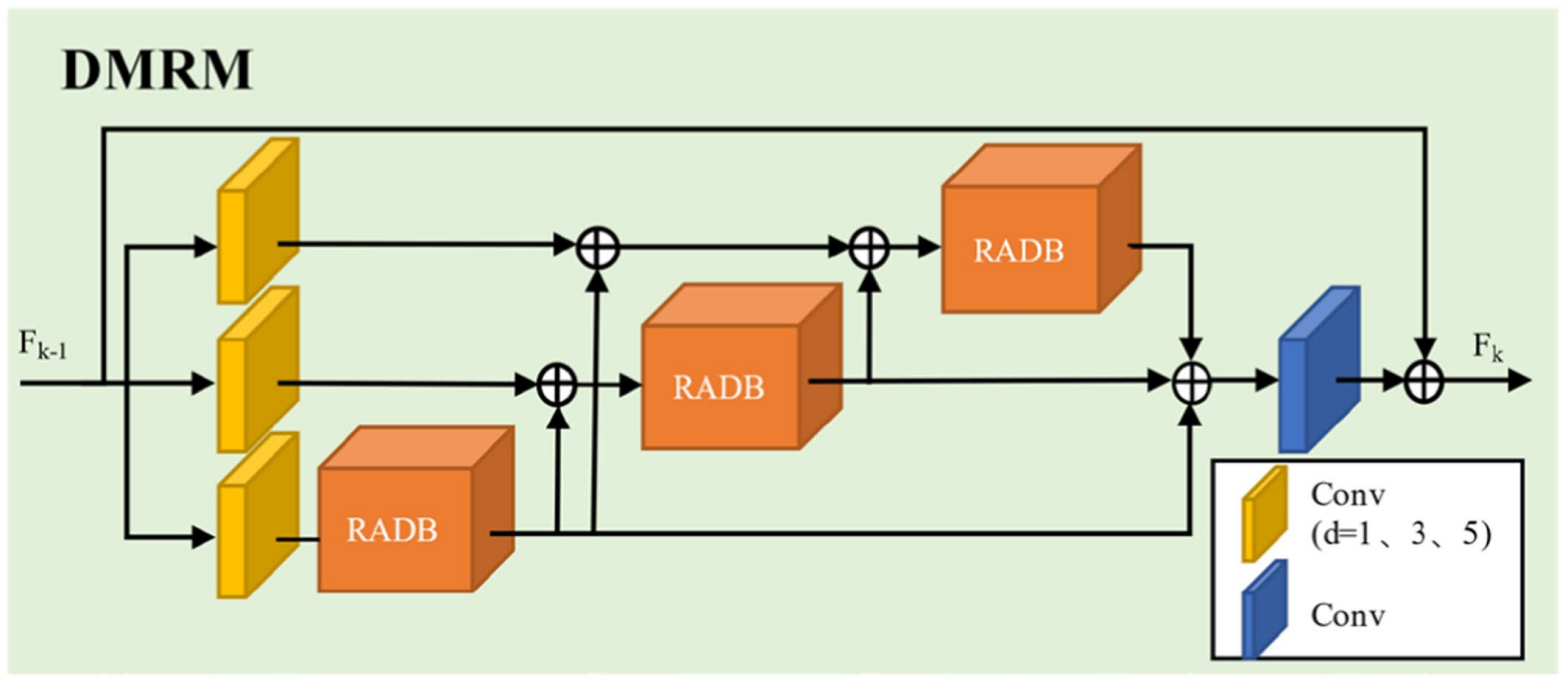
The structure of dilated multi-scale residual module.

**Figure 5:**
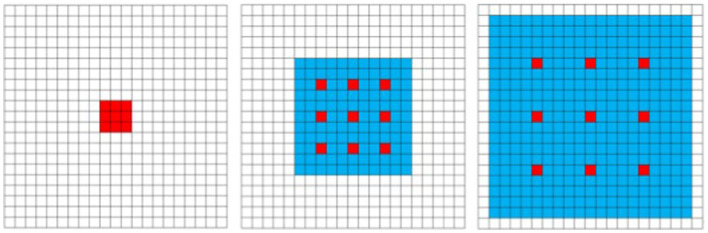
Schematic diagram of spatiotemporal convolution receptive field with dilation rate = 1, 3, 5.

Convolutional operations with convolution kernels of different sizes can extract multi-scale features of images. Based on this, this paper proposes a Dilated Multi-Scale Residual Module (DMRM) to fully learn image features, as shown in Figure 4. Specifically, we parallelly adopt dilated convolution with expansion rates of 1, 3, and 5 (as shown in Figure 5) to learn multi-scale features of images firstly. And dilated convolution can expand the receptive field without generating a large number of parameters. Then, we use the designed Residual Attention-Dense Block (See 2.3 for details) on each branch to further learn image features and gradually add residual connections to enhance model performance. Finally, we employ convolution and residual concatenation operations to further learn features.

### 3.3 Residual attention-dense block

**Figure 6:**
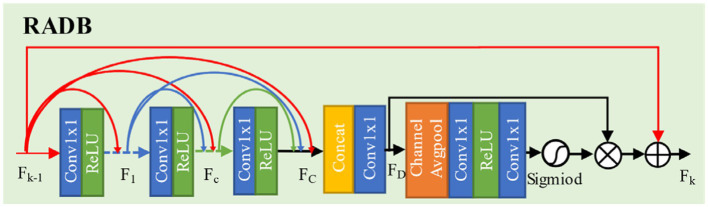
The structure of attention dense residual block.

To address issues such as insufficient feature extraction and loss of details in low-resolution images, this paper designs an Residual Attention Dense Residual Block (RADB), as shown in Figure 6.

This module consists of a Dense Residual Block (DRB) and a Channel Attention (CA). Firstly, we use three basic blocks composed of convolution and ReLU activation function to form dense residual block and to obtain feature map $F_{C}$. Subsequently, through the concatenation and a 1×1 convolution operation, the features extracted by each convolutional layer are merged and the channel data is simplified. It generates feature map $F_{D}$. Finally, we use channel attention to learn the features of different channels, while utilizing residual connections to enhance model performance.

Let the input and output of the RADB be denoted as $F_{k-1}$ and $F_{k}$, respectively. The dense residual block can be expressed by Equation 5:


(5) $$F_{c}=\sigma \left(W_{c}\left[F_{k-1},\;F_{1},\cdots ,\;F_{c-1}\right]\right)$$

Here, $\left[F_{k-1},\;F_{1},\cdots ,\;F_{c-1}\right]$ represents the concatenation of feature maps, producing $G_{0}+\left(c-1\right)\times G$ feature maps (where $W_{c}$ is the growth rate, set to 32 in this paper), $W_{c}$ is the weight of the convolutional layer, σ is the operation of the ReLU function, and $F_{c}$ is the feature map after the convolutional layer. The extracted features from each layer are then fused, with the result shown in Equation 6:


(6) $$F_{D}=H_{D}\left(\left[F_{k-1},\;F_{1},\cdots ,\;F_{c},\cdots F_{C}\right]\right)$$

Here, $H_{D}$ represents the convolution operation with a kernel size of 1, and $F_{D}$ denotes feature fusion. The fused features are then fed into the channel attention module, where they undergo adaptive average pooling followed by data dimensionality reduction, reducing the dimension of the fused features to their original size by 1/r(where r is the dimension compression ratio, taken as r=16). Subsequently, the features are processed through ReLU and Sigmoid function operations. Finally, the resulting feature information is fused with the original feature information, and this process is represented by Equations 7–9:


(7) $$\begin{array}{l} z=H_{GP}\left(F_{D}\right)\\ =\frac{1}{H\times W}\sum_{i=1}^{H}\sum_{j=1}^{W}F_{D}\left(i,j\right) \end{array}$$

(8) $$s=f_{\text{sigmoid}}\left(W_{\text{UP}}\delta \left(W_{\text{DOWN}}z\right)\right)$$

(9) $$F_{k}=F_{k-1}+s\cdot F_{D}$$

Here, $H_{GP}$ represents adaptive average pooling, $f_{\text{sigmoid}}$ denotes the operation of the Sigmoid function, $W_{\text{UP}}$ and $W_{\text{DOWN}}$ represent the weights of the channel upsampling and downsampling layers respectively, s indicates the result of the Sigmoid function operation, and $F_{k}$ represents the final output.

## 4 Experimental results and analysis

### 4.1 Datasets

This paper selects the publicly available DIV2K (Timofte et al., 2017) dataset for training, which contains 800 training images. The low-resolution (LR) images used for training are obtained by bicubic downsampling of high-resolution (HR) images. Data augmentation is performed using rotations of 90°, 180°, 270°, and horizontal flipping. The LR images are cropped into image blocks of size 32×32, and the HR images are also cropped into blocks of size 32s×32s, where s represents the scaling factor.

To evaluate the effectiveness of the model proposed in this paper, four widely used benchmark datasets are utilized for model performance assessment: Set5 (Zeyde et al., 2010), Set14 (Kingma and Ba, 2014), BSDS100 (Arbelaez et al., 2010), and Urban100 (Huang et al., 2015). Among these, the Set5 and Set14 datasets contain images of animals and plants; the BSDS100 dataset contains images of urban architecture, which have abundant edge information and pose greater reconstruction challenges.

### 4.2 Experimental environment and parameter settings

The network training platform used is Ubuntu 18.04, with the programming framework being Pytorch 1.2. The processor is an Intel Core i9-9900K, and the graphics card is an RTX 2080Ti with 11G of video memory; system memory is 64G. The network utilizes the Adam (Kingma and Ba, 2014) algorithm for optimization, with the momentum decay rates set to u=0.9 and v=0.99, step size η=0.001, and a numerically stable small constant $\varepsilon =10^{-8}$. The reasons for choosing η=0.001 are: A learning rate of 0.001 typically strikes a good balance between convergence speed and stability. This value is sufficiently high to ensure rapid initial learning, yet low enough to prevent significant oscillations or divergence during training. The reasons for choosing u=0.9 are: The default value of 0.9 provides a reasonable balance between considering recent gradient information and long-term trends. This value helps the optimizer effectively capture gradient directions while maintaining robustness against noisy updates. The reasons for choosing v=0.99 are: A high value such as 0.999 ensures that the second moment estimates (which capture gradient variance) are stable and less sensitive to short-term fluctuations. This helps maintain consistent update step sizes and prevents the optimizer from making overly aggressive updates. The reasons for choosing $\varepsilon =10^{-8}$ are: A small ε value is used to prevent division by zero during parameter update steps. This ensures numerical stability without significantly affecting the optimizer’s behavior. The overall network loss function is governed by the $L_{1}$ function. The entire network is trained for 100 epochs, with a learning rate of 0.0001 and a batch size of 32.

### 4.3 Evaluation criteria

This paper utilizes two objective evaluation metrics to verify experimental results: Peak Signal to Noise Ratio (PSNR; Fei et al., 2007) and Structural Similarity (SSIM; Wang et al., 2004). The calculation method for PSNR is as follows (Equations 10, 11):


(10) $$MSE=\frac{1}{H\times W}\sum_{i=1}^{H}\sum_{j=1}^{W}{\left(X\left(i,j\right)-Y\left(i,j\right)\right)}^{2}$$

(11) $$PSNR=10\times \mathrm{lg}\frac{{\left(2^{n}-1\right)}^{2}}{MSE}$$

Where MSE represents the mean squared error between the current image X(i,j) and the reference image Y(i,j), H and W are the height and width of the image respectively, n is the number of bits per pixel, typically 8. PSNR is measured in dB, where a higher value indicates less distortion and better reconstruction quality.

SSIM is also a measure of image quality, evaluating the reconstruction effect of images from three aspects: brightness, contrast, and structure. Its calculation formula is as follows (Equation 12):


(12) $$\text{SSIM}=\frac{\left(2\mu_{f}\mu_{\hat{f}}+C_{1}\right)\left(\sigma_{f\hat{f}}+C_{2}\right)}{\left(\mu_{f}^{2}+\mu_{\hat{f}}^{2}+C_{1}\right)\left(\sigma_{f}^{2}+\sigma_{\hat{f}}^{2}+C_{2}\right)}$$

Where f represents the real high-resolution image, $\overset{\wedge }{f}$ represents the reconstructed high-resolution image, $\mu_{f}$ and $\mu_{\hat{f}}$ represent the average grayscale values of the real and reconstructed high-resolution images, $\sigma_{f}$ and $\sigma_{\hat{f}}$ respectively denote the variances of the real and reconstructed high-resolution images, $\sigma_{f\hat{f}}$ represents the covariance between the real and reconstructed high-resolution images, $C_{1}$ and $C_{2}$ are constants.

### 4.4 Comparative experiments

#### 4.4.1 Objective result evaluation

To thoroughly validate the effectiveness and superiority of the proposed algorithm, it was compared with seven other super-resolution algorithms: Bicubic, SRCNN (Dong et al., 2014), FSRCNN (Chao et al., 2015), VDSR (Timofte et al., 2017), DRCN (Kim et al., 2016), LapSRN (Lai et al., 2017), MemNet (Tai et al., 2017), and DSRNet (Tian et al., 2023). The reconstruction results were evaluated on four standard test sets: Set5, Set14, BSDS100, and Urban100, with magnification factors of 2×, 3×, and 4×.

The comparison results are presented in Table 1, where bold indicates the best results and underlined values denote the second-best results. Through numerical comparison, it is evident that the proposed algorithm achieves significantly higher average PSNR and SSIM values compared to other state-of-the-art methods. Specifically, on the Set14 dataset, compared to the second-best results, the proposed algorithm demonstrates an improvement of 0.25 dB, 0.06 dB, and 0.01 dB in PSNR for magnification factors of 2×, 3×, and 4×, respectively. On the Urban100 dataset, compared to the second-best results, the proposed algorithm achieves a PSNR improvement of 0.67 dB, 0.13 dB, and 0.03 dB for magnification factors of 2×, 3×, and 4×, respectively. Through horizontal comparison, we found that as the magnification factor increases, the reconstruction effectiveness of our algorithm on the Set5 dataset becomes increasingly pronounced. This suggests that our method is particularly well-suited for reconstructing images of both portraits and natural landscapes.

**Table 1:** Comparison of reconstruction results under baseline data.

Method	Scale	Set5	Set14	BSDS100	Urban100
		PSNR/SSIM	PSNR/SSIM	PSNR/SSIM	PSNR/SSIM
Bicubic	2	33.66/0.9299	30.24/0.8688	29.56/0.8431	26.88/0.8403
SRCNN	2	36.66/0.9524	32.45/0.9067	31.36/0.8879	29.50/0.8946
FSRCNN	2	37.00/0.9559	32.75/0.9098	31.51/0.8939	29.88/0.9020
VDSR	2	37.53/0.9587	33.03/0.9124	31.90/0.8960	30.77/0.9140
DRCN	2	37.63/0.9588	33.08/0.9118	31.85/0.8942	30.75/0.9133
LapSRN	2	37.52/0.9591	33.08/0.9130	31.08/0.8950	30.41/0.9101
MemNet	2	37.78/0.9597	33.28/0.9142	32.08/0.8978	31.31/0.9195
DSRNet	2	37.61/0.9584	33.30/0.9145	31.96/0.8965	31.41/0.9209
Ours	2	**37.98/0.9606**	**33.55/0.9179**	**32.11/0.8989**	**32.08/0.9278**
Bicubic	3	30.39/0.8682	27.55/0.7742	27.21/0.7385	24.46/0.7349
SRCNN	3	32.75/0.9090	29.28/0.8209	28.41/0.7863	26.24/0.7989
FSRCNN	3	33.18/0.9140	29.37/0.8240	28.50/0.7937	26.41/0.8161
VDSR	3	33.98/0.9212	29.77/0.8314	28.82/0.7976	27.14/0.8279
DRCN	3	33.82/0.9226	29.76/0.8311	28.80/0.7963	27.15/0.8276
LapSRN	3	33.16/0.9140	29.43/0.8242	28.53/0.7910	27.43/0.8080
MemNet	3	34.09/0.9248	30.00/0.8350	28.96/0.8001	27.56/0.8376
DSRNet	3	33.92/0.9227	30.10/0.8378	28.90/0.8003	27.63/0.8402
Ours	3	**34.12/0.9249**	**30.16/0.8410**	**28.97/0.8021**	**27.76/0.8410**
Bicubic	4	28.42/0.8104	26.00/0.7027	25.96/0.6675	23.14/0.6577
SRCNN	4	30.48/0.8628	27.49/0.7503	26.90/0.7101	24.52/0.7221
FSRCNN	4	30.71/0.8657	27.59/0.7535	26.98/0.7150	24.62/0.7280
VDSR	4	31.35/0.8838	28.01/0.7674	27.29/0.7251	25.18/0.7524
DRCN	4	31.53/0.8854	28.02/0.7670	27.23/0.7233	25.14/0.7510
LapSRN	4	31.54/0.8811	28.19/0.7720	27.32/0.7280	25.21/0.7560
MemNet	4	31.74/0.8893	28.26/0.7723	27.40/0.7281	25.50/0.7630
DSRNet	4	31.71/0.8874	28.38/0.7760	27.43/0.7303	25.65/0.7693
Ours	4	**31.96/0.8931**	**28.39/0.7820**	**27.49/0.7343**	**25.68/0.7730**

#### 4.4.2 Subjective effect evaluation

**Figure 7:**
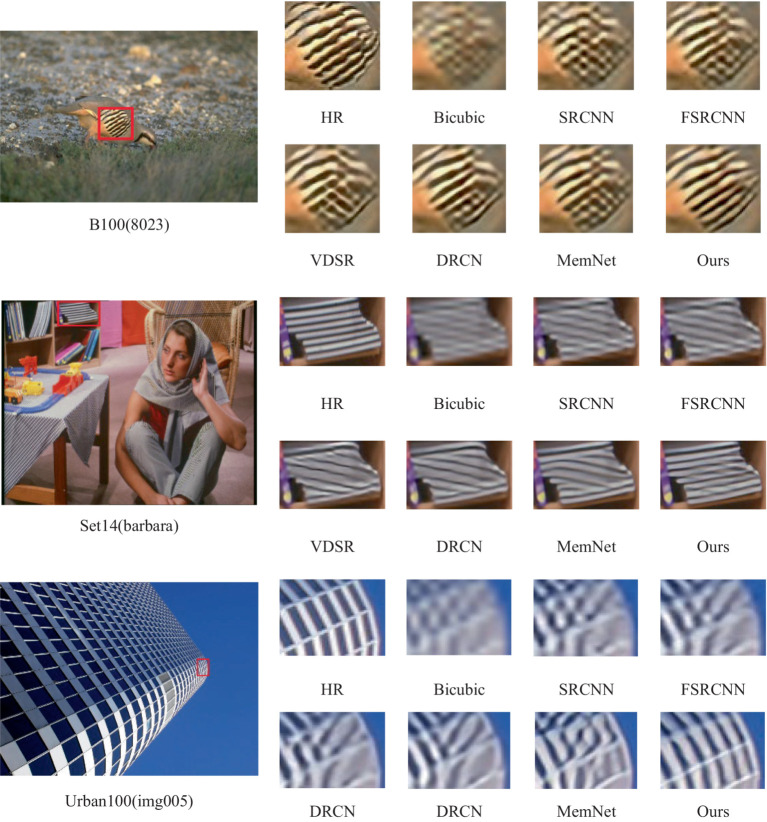
Comparison of visual effects at 4 × magnification under the standard test set. Adapted with permission from “On Single Image Scale-Up Using Sparse-Representations” (https://link.springer.com/chapter/10.1007/978-3-642-27413-8_47) and Github address: https://github.com/jbhuang0604/SelfExSR?tab=readme-ov-file#introduction.

Further subjective evaluation of the visual effects is conducted. Figure 7 presents the visual reconstruction results of the proposed algorithm and other comparative algorithms at a 4× magnification factor on the Set14, BSDS100, and Urban100 datasets.

For the image “barbara” in the Set14 dataset, the reconstruction images produced by other comparative algorithms exhibit severe blurriness, making it difficult to distinguish between adjacent edges of books. In contrast, the images reconstructed by the proposed algorithm can clearly discern the edges between adjacent books; For the image “8,023” in the BSDS100 dataset, the textures between bird feathers in the reconstruction images produced by other comparative algorithms vary in degrees of blurriness. However, the proposed algorithm almost perfectly restores the textures between bird feathers; For the image “img005” in the Urban100 dataset, in the area at the top of the building, compared to the reconstruction quality of MemNet, the images reconstructed by the proposed algorithm not only avoid geometric distortions but also construct more regular textures.

The superior reconstruction capability of the proposed method is attributed to the RADB and DMRM. The RADB effectively extracts similar features between images, while the DMRM comprehensively integrates image information between feature maps, preserving more high-frequency information.

### 4.5 Ablation study

To ensure the fairness of the experiments, all training batches were conducted for 400 epochs, and the average PSNR values for a scaling factor of 4 on the Set5 dataset were compared. The best results are highlighted in bold.

#### 4.5.1 The impact of dilated convolutions and the RADB module

To verify the effectiveness of dilated convolutions and the RADB module, we compared the proposed algorithm with versions of the algorithm that excluded the dilated convolutions and the RADB module, respectively. As shown in Table 2, the PSNR value without dilated convolutions and the RADB module was 31.42 dB. When using dilated convolutions, the PSNR value increased to 31.68 dB, representing an improvement of 0.26 dB. With the addition of the RADB module, the PSNR value increased to 31.81 dB, an improvement of 0.39 dB. When both dilated convolutions and the RADB module were used, the PSNR value increased to 31.96 dB, an improvement of 0.54 dB. This demonstrates that the dilated convolutions and RADB module used in this study effectively extract feature information, significantly enhancing the network’s learning ability.

**Table 2:** The impact of dilated convolutions on reconstruction performance.

Algorithm		PSNR (dB)
Dilation convolution	RADB	
		31.42
	✓	31.81
✓		31.68
✓	✓	**31.96**

#### 4.5.2 The impact of dilated convolution kernels

To verify the effectiveness of selecting different dilated convolution kernels, we compared kernels of sizes 1, 3, 5 with kernels of sizes 1, 1, 1; 3, 3, 3; and 5, 5, 5. The results are shown in Table 3. From Table 3, it can be concluded that the kernel sizes of 1, 3, 5 are optimal. This study differs from previous super-resolution algorithms that use dilated convolutions to achieve a large receptive field. To avoid the drawback of not fully covering all pixels, this study adjusts the dilation rates to nearly fully cover the receptive field, thereby allowing the network to achieve a larger perceptual field. This helps the algorithm to extract non-local similar features and restore clear images.

**Table 3:** The impact of different dilated convolution kernels on reconstruction performance.

Dilation rate	PSNR
1,1,1	31.54
3,3,3	31.85
5,5,5	31.47
1,3,5	**31.96**

#### 4.5.3 Assessment of LPIPS indicators

To further illustrate the effectiveness of the proposed algorithm, we compared its super-resolution reconstruction results at different scales with those of DRCN, LapSRN, and MemNet on the Set5, Set14, BSD100, and Urban100 test datasets using the Learned Perceptual Image Patch Similarity (Zhang et al., 2018) (LPIPS) evaluation metric, as shown in Table 4. LPIPS is primarily used to measure the difference between two images and is more aligned with human perception compared to traditional methods such as PSNR and SSIM. A lower LPIPS value indicates greater similarity between the two images, while a higher value indicates a larger difference.

**Table 4:** Average LPIPS values of different SR algorithms.

Method	Scale	Set5	Set14	BSD100	Urban100
		LPIPS	LPIPS	LPIPS	LPIPS
DRCN	2	0.0563	0.0946	0.1471	0.0678
LapSRN		0.0566	0.0943	0.1442	0.0642
MemNet		0.0551	0.0924	0.1436	0.0615
Ours		**0.0548**	**0.0898**	**0.1398**	**0.0597**
DRCN	3	0.1259	0.2090	0.2824	0.1577
LapSRN		0.1260	0.2082	0.2820	0.1564
MemNet		0.1241	0.2074	0.2808	0.1547
Ours		**0.1218**	**0.2009**	**0.2760**	**0.1466**
DRCN	4	0.1761	0.2893	0.3774	0.2365
LapSRN		0.1752	0.2881	0.3768	0.2336
MemNet		0.1714	0.2841	0.3710	0.2235
Ours		**0.1707**	**0.2815**	**0.3685**	**0.2186**

As shown in Table 4, the proposed algorithm achieves the best LPIPS evaluation results for super-resolution at different scales on the Set5, Set14, BSD100, and Urban100 test datasets. For example, with a scaling factor of 2, the LPIPS values of the proposed algorithm are lower by 0.0003, 0.0006, 0.0008, and 0.0018, respectively, compared to the second-best results. This indicates that the images reconstructed by the proposed algorithm are more aligned with human perception, exhibiting better perceptual quality and minimal distortion.

## 5 Conclusion

This paper proposes a super-resolution reconstruction algorithm based on dilated convolution for addressing issues such as limited receptive field, insufficient multi-scale feature extraction, and loss of image feature information in the process of image super-resolution reconstruction. The algorithm introduces an residual attention-dense block, which employs dense residual block and channel attention to fully learn the features of the original low resolution images. In addition, this paper proposes the dilated multi-scale residual module to extract multi-scale features, using dilated convolutions with different expansion rates. Additionally, a residual nested network is utilized to fully exploit image features at different depths, leading to significant improvements in super-resolution performance. Experimental results demonstrate that the proposed algorithm outperforms other super-resolution algorithms such as Bicubic, SRCNN, ESPCN, VDSR, DRCN, LapSRN, MemNet and DSRNet.

## Data availability statement

The original contributions presented in the study are included in the article/supplementary material, further inquiries can be directed to the corresponding author.

## Author contributions

SW: Conceptualization, Investigation, Methodology, Supervision, Writing – original draft, Writing – review & editing. MZ: Investigation, Software, Writing – original draft. MM: Data curation, Supervision, Writing – review & editing.
